# Supplementary material for: Field-free spin–orbit torque switching in ferromagnetic trilayers at sub-ns timescales
Source: Nat Commun. 2024 Feb 28;15:1814. doi: 10.1038/s41467-024-46113-1 (PMC10901790; doi:10.1038/s41467-024-46113-1)
Supplement: Supplementary file 1 — Supplementary Information [file 41467_2024_46113_MOESM1_ESM.pdf]

Supplementary Note 1: DC induced field-free switching with different Ti thicknesses

Direct current (DC) induced field-free SOT switching has been realized in CoFeB (4 nm)/Ti (t_{Ti} nm)/CoFeB (1 nm)/MgO (3.2 nm)/Ta (2 nm) structures with t_{Ti} from 1 nm to 4 nm as shown in Fig. 1c and Supplementary Fig. 1. In most cases with FM trilayers, field-free magnetization switching of ~75% can be achieved. However, this switching ratio can be notably enhanced ranging from 80% to 100%, by employing a post-pillar etching step where the device is covered with a layer of Si_3N_4. We believe that this post-pillar etching process reduces the edge effects, resulting in the SOT-induce full magnetization switching without an external magnetic field.

The obtained results show that the switching polarity remains the same for Ti thicknesses up to 4 nm, which aligns with the findings of the previous report[1]. Besides, there is no field-free SOT switching in the CoFeB/Ti/CoFeB sample with a thicker CoFeB (> 6 nm). Moreover, no field-free SOT switching occurs in samples with a large t_{Ti} (> 4 nm). This indicates that the out-of-plane SOT responsible for field-free switching is mainly generated by the CoFeB (4 nm)/Ti interface. It is found that the t_{Ti} = 3 nm sample shows the most stable switching performance and lowest switching current density (~12 MA cm^{-2}) among all Ti thicknesses.

Concerning the spin-Hall current originating from the bottom Ta layer, it is important to note that this in-plane SOT is primarily influenced by an applied magnetic field[2]. However, in the absence of an external field, in-plane SOT cannot switch the magnetization direction alone without out-of-plane SOT[1]. Our micromagnetic simulations also have confirmed that in-plane SOT alone (Fig. 4a, η=0.1°) is insufficient for achieving PMA switching. This suggests that the out-of-plane spins are the main contributor of field-free switching in the magnetic trilayer.

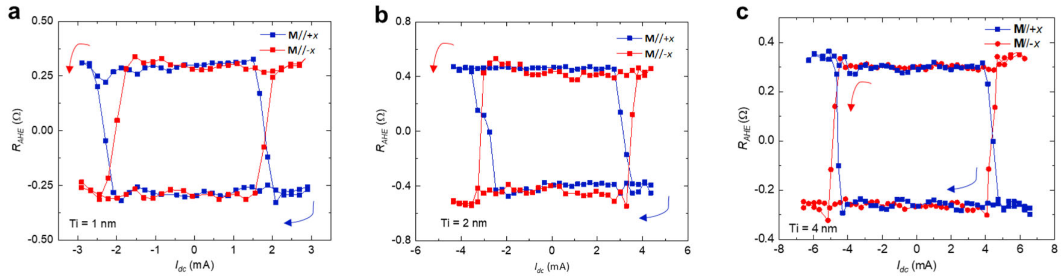

Supplementary Figure 1 | Field-free deterministic SOT switching as a function of current amplitude at different t_{Ti}, with **M** saturated along the +*x* and –*x* direction. The devices have a pillar radius (*R*) of 500 nm and a channel width of 1.8 μm. J_c is 14.2 MA cm^{-2} for t_{Ti} = 1 nm (**a**), 15.7 MA cm^{-2} for t_{Ti} = 2 nm (**b**), and 32.5 MA cm^{-2} for t_{Ti} = 4 nm (**c**).

Supplementary Note 2: Confirmation of *z* spins by current induced anomalous Hall loop shift

Out-of-plane SOT effective field can be characterized by the shift (ΔH_{shift}) of the anomalous Hall effect (AHE) loop under different I_{dc}[3,4]. Supplementary Fig. 2 shows the magnetic hysteresis loops of t_{Ti} = 3 nm trilayer sample when I_{dc} = ±0.1 mA (**M**//-*x*), ±2.5 mA (**M**//-*x*) and ±2.5 mA (**M**//+*x*). The center of the hysteresis loop is defined as $H_{\text{center}} = (H_+ - H_-)/2$, where H_+ and H_- refer to the positive and negative magnetization reversal field, respectively. The loop shift $\Delta H_{shift} = H^{+I}_{center}$-$H^{-I}_{center}$, where +I and –I are positive and negative I_{dc}. Based on the AHE loops under different I_{dc}, the existence of out-of-plane spin can be confirmed experimentally.

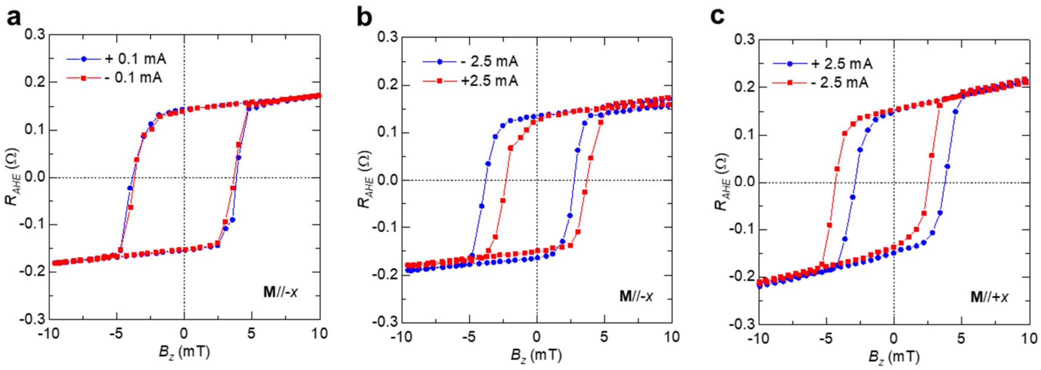

Supplementary Figure 2 | Loop shift measurement under direct currents in a *R* = 2 μm pillar device. a, AHE loops with I_{dc}= ±0.1 mA (**M**//-*x*). **b**, I_{dc}= ±2.5 mA (**M**//-*x*). **c**, I_{dc}= ±2.5 mA (**M**//+*x*).

Supplementary Note 3: Calibration of the equivalent J_c under a short pulse

Supplementary Fig. 3a shows the calibration setup for the true value of the pulse current density on the pillar device. We use the PSPL 10060A pulse generator to generate a square voltage pulse with τ_p ranging from 0.1 to 10 ns. Tektronix DPO72004C oscilloscope is used to detect the pulse voltage amplitude before (V_1) and after (V_3) the device channel. It has been reported that the real applied voltage on the device (V_{DUT}) can be evaluated by $V_{\mathrm{DUT}} = 2V_{\mathrm{in}}(1\text{-}L)R_{\mathrm{DUT}}/(R_{\mathrm{DUT}} + 50\ \Omega) = 2V_{\mathrm{m}}R_{\mathrm{DUT}}/(R_{\mathrm{DUT}} + 50\ \Omega) \approx 2V_{\mathrm{m}}\ (R_{\mathrm{DUT}} \gg 50\ \Omega)$[5]. Where V_{in} is the input pulse amplitude, L is the system loss coefficient, and V_{m} is the measured value by the oscilloscope. The accurate V_{DUT} is about 2 times larger than that of the V_{m}. The real voltage on our trilayer device $V = V_1 + V_2 = 2V_1 - V_3\ (R \gg 50\ \Omega)$[6]. Where V_1 is the pulse voltage after the bias tee which can be detected by the oscilloscope when disconnecting the device, V_2 is the reflected voltage, and V_3 is the transmission voltage which can be detected by oscilloscope when connecting device. The transmission and system loss change with the frequency and

can be reflected by the calibration of the effective voltage factor ($V_{\text{eff, factor}}$) under various τ_p as shown in Supplementary Fig. 3c. The equivalent J_c then can be determined considering the device resistance R and device dimension.

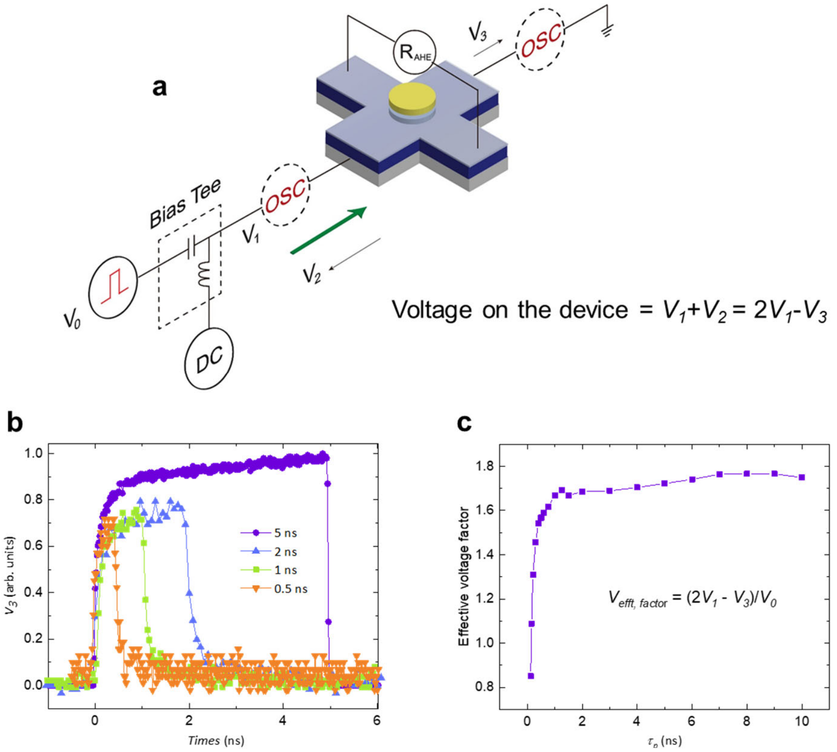

Supplementary Figure 3 | Determination of the effective pulse voltage on the pillar device. a, Measurement setup for the effective voltage calibration. **b**, Transmission voltage with different τ_p. **c**, Effective voltage factor as a function of τ_p, where V_0 is the ideal output of the pulse generator.

Supplementary Note 4: Reproducibility test for t_{Ti} = 3 nm trilayer samples

We have demonstrated repetitive field-free switching in a single device (R = 300 nm) without subsequent saturation of the bottom FM layer. This is evident in Supplementary Fig. 4a, which shows the sustained stable switching behavior even after more than one thousand cycles. We have also investigated switching behaviors across multiple devices

and have observed consistent outcomes, as shown in Supplementary Fig. 4b,c. Notably, we found that devices made from films with identical layer thicknesses maintain their reproducibility, even from different film batches.

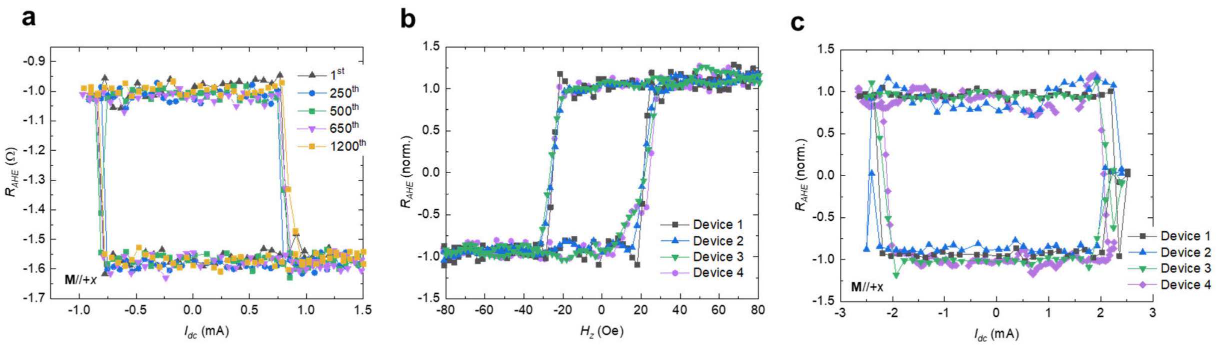

Supplementary Figure 4 | a, Repetitive field-free switching in a single device (*R* = 300 nm, *W* = 800 nm). **b**, AHE loops. **c**, Field-free current switching across multiple devices.

Supplementary Note 5: Repeated field-free short pulse switching in another *R* = 500 nm device

Supplementary Fig. 5 show the short pulse switching data for t_{Ti} = 3 nm obtained from another pillar device (device 2, *R* = 500 nm). Similar to the findings for **M**//-*x* shown in Fig. 2, asymmetry has been also observed when applying both positive and negative pulses. The magnetic symmetry is broken and the domain propagation is depending on the sign of the magnetization, current and external field[7] (discussed in Supplementary Note 7). To establish one dataset in one device, we repeated these measurements 5 times.

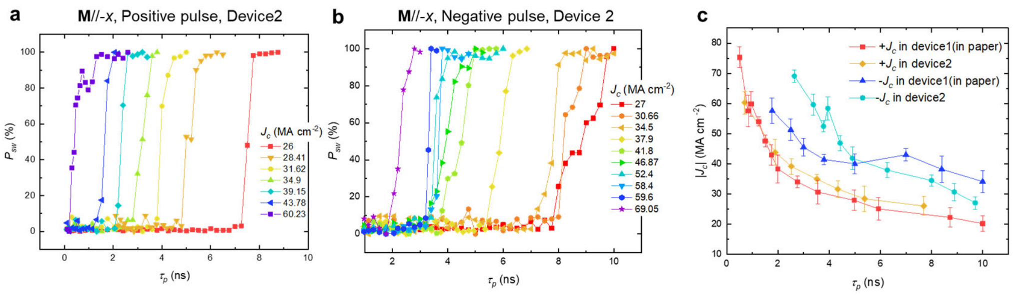

Supplementary Figure 5 | a, **b**, Short pulse field-free SOT switching probability as a function of (**a**) positive and (**b**) negative pulse duration at t_{Ti} = 3 nm from another pillar device (R = 500 nm) when **M**//-x. **c**, $|J_c|$ for P_{sw} = 0.9 in two devices.

Supplementary Note 6: Field-free short pulse switching across various dimensions

Short pulse switching was performed in devices with varying R, ranging from 75 to 550 nm with **M**//+x. As the device size decreases, the influence of edges and interfaces becomes more significant, resulting in a considerable increase in J_c and writing energy, as shown in Supplementary Fig. 6e,f. In smaller devices, the switching behavior becomes stochastic with small writing currents (Supplementary Fig. 6c).

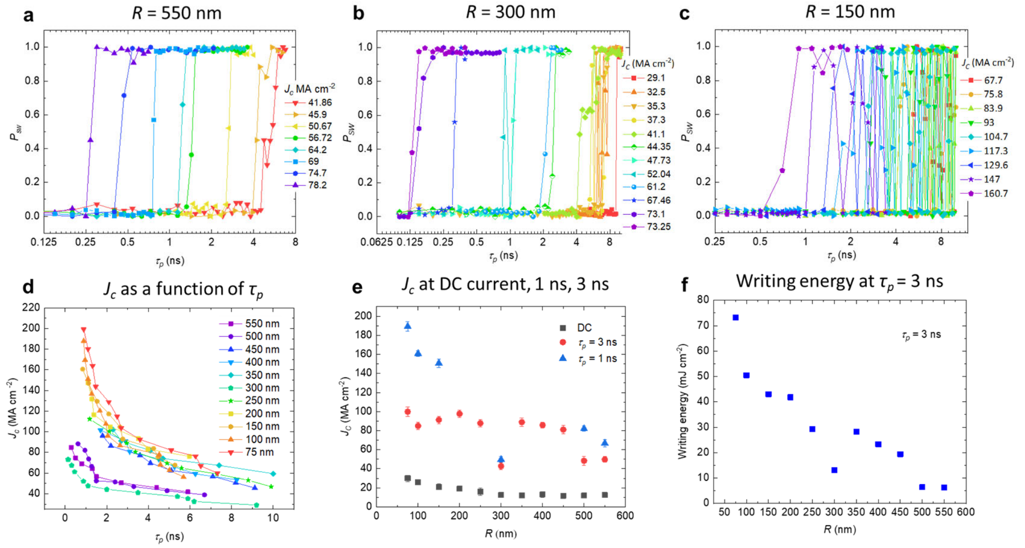

Supplementary Figure 6 | Short pulse field-free SOT switching probability with (**a**) R = 550 nm, (**b**) R = 300 nm, and (**c**) R = 150 nm. **d**, J_c for P_{sw} = 0.9 as a function of τ_p with varying R. **e**, J_c as a function of R with DC, τ_p = 1 ns, 3 ns. **f**, Writing energy as a function of R.

Supplementary Note 7: J_c as a function of $1/\tau_p$ with varying R and the linear fittings

We plotted $1/\tau_p$ versus J_c based on Supplementary Fig. 6d and show the results within 2.5 GHz in Supplementary Fig. 7. Upon comparison with the results in Fig. 3b in the main text, it is observed that as the dimensions decrease, the linearity improves. This could be attributed to domain wall switching becoming closer to a single domain behavior at smaller dimensions, as assumed in the theoretical model[8,9].

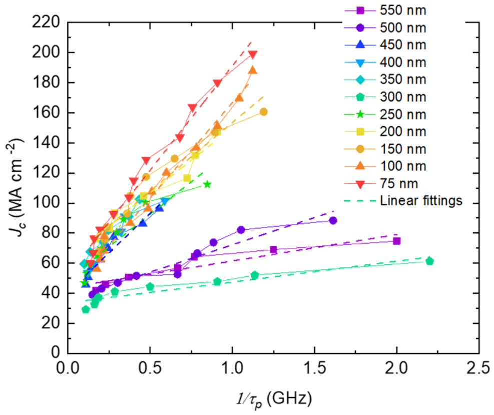

Supplementary Figure 7 | J_c for P_{sw} = 0.9 as a function of $1/\tau_p$ with varying R.

Supplementary Note 8: Field-free SOT short pulse switching probability with different Ti thicknesses when M//+*x*

As shown in Supplementary Fig. 8a,b, sub-ns SOT switching can also be achieved at t_{Ti} = 1 nm without any assisted field and the minimum pulse width required to achieve P_{sw} = 0.9 can be as short as 0.76 ns for J_c = 59.14 MA cm^{-2}. When τ_p is around 3 ns, J_c is 33 MA cm^{-2} to achieve P_{sw} = 0.9 for t_{Ti} = 1 and 3 nm samples, while J_c increases to 42 MA cm^{-2} for t_{Ti} = 2 nm. The Ti thickness may impact the interlayer coupling and interfacial spin-orbit field which may further influence the energy barrier and the switching efficiency.

Short pulse field-free SOT switching probabilities for t_{Ti} = 3 nm when **M**//+*x* have been shown in Supplementary Fig. 8c,d. Compared to the results of **M**//-*x* shown in Fig. 2, there can be some differences in τ_p at certain switching current densities. Sample geometry can be a possible cause of this asymmetry as the etching process can unavoidably induce device asymmetry and the asymmetric domain nucleation takes place at the edge of the sample.

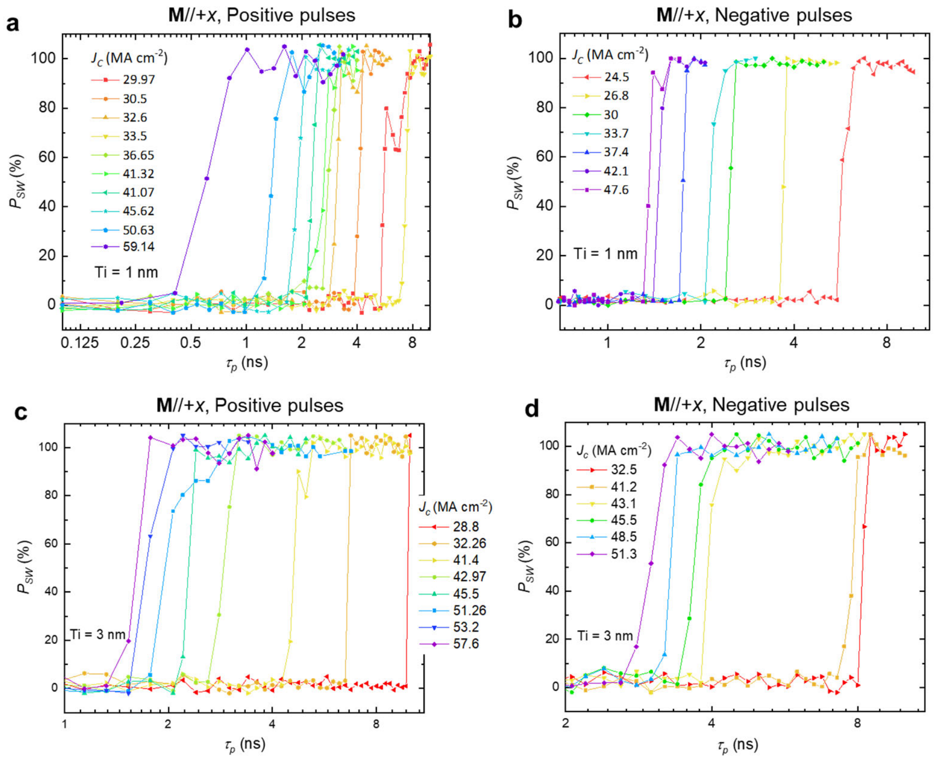

Supplementary Figure 8 | Short pulse field-free SOT switching probability as a function of pulse duration when M//+*x*. a,**b**, Pulse switching probability (P_{sw}) for t_{Ti} = 1 nm with positive pulses (**a**) and negative pulses (**b**). **c**,**d**, P_{sw} for t_{Ti} = 3 nm with positive pulses (**c**) and negative pulses (**d**).

Supplementary Note 9: Switching probabilities as a function of current density

Supplementary Figure 9 are the switching probabilities as a function of write current at different pulse widths for t_{Ti} = 3 nm. In DC switching, the current density tends to be relatively low as the spin torque accumulates gradually over an extended period. On the

other hand, short pulse switching demands higher current densities as the pulse width decreases to the sub-ns region.

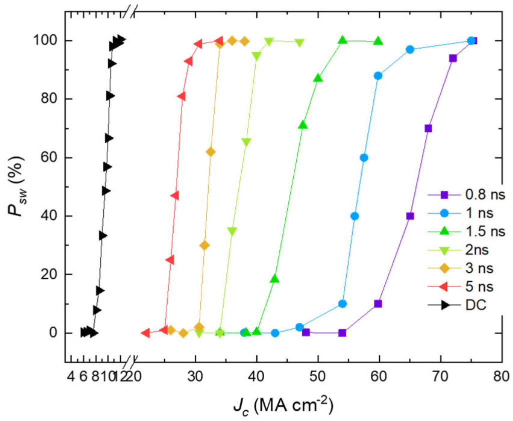

Supplementary Figure 9 | DC and short pulse induced field-free SOT switching probabilities as a function of current density for t_{Ti} = 3 nm. The device with R = 500 nm is the same one in Fig. 1c and Fig. 2a.

Supplementary Note 10: Parameter derivation of the switching probability

It has been studied that the full switching probability distributions can be derived by[9,10]

$$\mathrm{P} = \exp\left\{-4\xi exp\left[-\frac{2\tau(\frac{I}{I_0}-1)}{\tau_D}\right]\right\}$$

Where ξ is the energy barrier and τ_D is the characteristic time associated with the switching dynamics, and we use τ_D to reflect the incubation time. Typical experimental and fitting results are shown in Supplementary Fig. 10, and the extracted τ_D corresponding to the switching probabilities in Fig. 2a are shown in Fig. 3c.

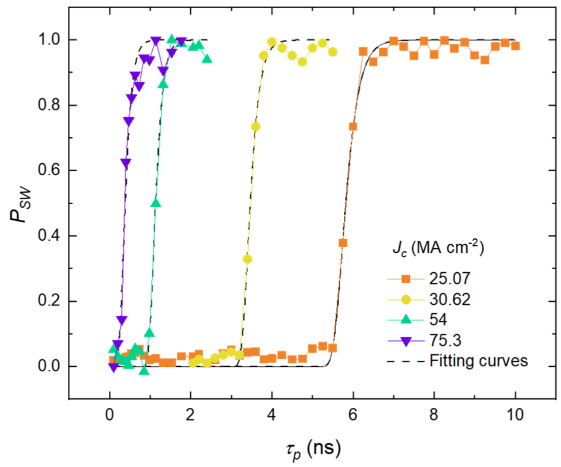

Supplementary Figure 10 | Experimental switching probability for t_{Ti} = 3 nm sample and the fitting curves.

Supplementary Note 11: Micromagnetic simulations of SOT switching for different spin-*z* polarization angles

We have performed micromagnetic simulations on Mumax3[11]. Here, the PMA layer thickness is 1 nm, the saturation magnetization M_s is 650 kA m^{-1}, the exchange stiffness constant is 2.0×10^{-11} J m^{-1}, the anisotropy energy is 3.0×10^{5} J m^{-3}, the effective damping-like torque (DLT) efficiency is 0.15, and the damping constant is 0.02. The spin polarization angle η is varied from 0 to 90 degree. The results (Supplementary Fig. 11) demonstrate that for pure *y*-spin (blue star, $\eta = 0°$), the symmetry-breaking magnetic field along the *x*-axis is essential for switching, with an estimated switching current (J_{sw}) of 46.5 MA cm^{-2}. In the case of *z*-spin (red dots, field-free), introducing a 5° spin-*z* polarization leads to a 30% reduction in J_{sw}, while a 10° spin-*z* polarization results in a 42.5% decrease in J_{sw}.

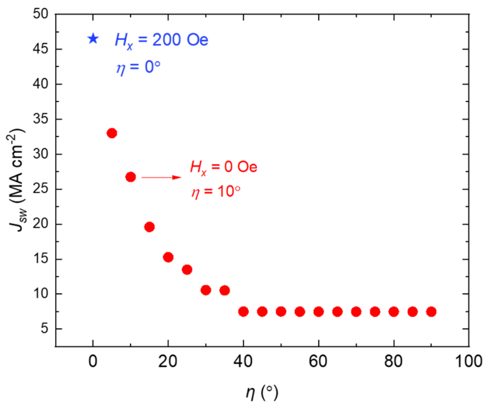

Supplementary Figure 11 | J_{sw} for different η at zero field (red dots). The blue star shows J_{sw} for η = 0° (pure *y*-spin) with the external magnetic field H_x = 200 Oe.

References

1 Choi, G. *et al.* Thickness Dependence of Interface-Generated Spin Currents in Ferromagnet/Ti/CoFeB Trilayers. *Adv. Mater. Interfaces* **9**, 2201317 (2022).

2 Ryu, J. *et al.* Efficient spin–orbit torque in magnetic trilayers using all three polarizations of a spin current. *Nat. Electron.* **5**, 217-223 (2022).

3 Yu, G. *et al.* Switching of perpendicular magnetization by spin–orbit torques in the absence of external magnetic fields. *Nat. Nanotechnol.* **9**, 548-554 (2014).

4 Pai, C.-F., Mann, M., Tan, A. J. & Beach, G. S. Determination of spin torque efficiencies in heterostructures with perpendicular magnetic anisotropy. *Phys. Rev. B* **93**, 144409 (2016).

5 Zeng, Z. *et al.* Effect of resistance-area product on spin-transfer switching in MgO-based magnetic tunnel junction memory cells. *Appl. Phys. Lett.* **98**, 072512 (2011).

6 Trueman, C. W. *Basic Demonstrations with BOUNCE*, <https://users.encs.concordia.ca/~trueman/bounce/demos.htm> (2001).

7 Baumgartner, M. *et al.* Spatially and time-resolved magnetization dynamics driven by spin–orbit torques. *Nat. Nanotechnol.* **12**, 980-986 (2017).

8 Garello, K. *et al.* Ultrafast magnetization switching by spin-orbit torques. *Appl. Phys. Lett.* **105**, 212401 (2014).

9 Bedau, D. *et al.* Spin-transfer pulse switching: From the dynamic to the thermally activated regime. *Appl. Phys. Lett.* **97**, 262502 (2010).

10 Liu, H. *et al.* Dynamics of spin torque switching in all-perpendicular spin valve nanopillars. *J. Magn. Magn. Mater.* **358**, 233-258 (2014).

11 Vansteenkiste, A. *et al.* The design and verification of MuMax3. *AIP Adv.* **4**, 107133 (2014).
